# Psychological Stress as a Mediator in the Relationships Between Personality Characteristics and Eye-Blinking Behavior

**DOI:** 10.3390/bs15111567

**Published:** 2025-11-17

**Authors:** Reut Ifrah, Avi Besser, Ayelet Goldstein, Yevgeny Beiderman, Liat Gantz

**Affiliations:** 1 Department of Optometry and Vision Science, Jerusalem Multidisciplinary College, Jerusalem 9101001, Israel; liatg@jmc.ac.il; 2 Department of Communication Disorders, Jerusalem Multidisciplinary College, Jerusalem 9101001, Israel; 3 Department of Computer Science, Jerusalem Multidisciplinary College, Jerusalem 9101001, Israel; ayeletgo@jmc.ac.il; 4 Faculty of Electric and Electronics Engineering, Holon Institute of Technology, Holon 5810201, Israel; beidermany@hit.ac.il

**Keywords:** personality, big-five, perceived stress, blinking behavior

## Abstract

This study explores the relationship between personality traits, perceived psychological stress, and blinking behavior. The research is grounded in the Big-Five personality model, particularly focusing on neuroticism, extraversion, and conscientiousness, and their associations with stress responses, and physiological markers such as spontaneous blink rate (SBR). Previous studies suggest that personality influences stress and physiological responses; however, the link to blinking behavior remains underexplored. The study hypothesized that neuroticism is positively associated with blinking variability, mediated by perceived stress, whereas extraversion and conscientiousness demonstrate negative associations. The study included 86 participants (74 females, mean age 21.94 ± 2.51 years, age range 18–31). Blink dynamics, expressed as blinks per minute, were measured during a sustained reading task on a laptop at a distance of 45 cm. Participants completed validated online questionnaires, including the Big-Five Inventory (BFI-2) and the Perceived Stress Scale (PSS-14). The relationships between blink rate and questionnaire outcomes were examined. Path analysis was used to test the hypothesized direct and indirect effects. Findings indicate that higher neuroticism correlates with increased perceived stress and an increased blink rate, supporting the mediation hypothesis. Additionally, conscientiousness was negatively associated with perceived stress, which in turn was related to reduced blinking. These findings suggest that personality traits influence physiological responses to stress, with potential implications for understanding stress-related behaviors and developing biomarkers for psychological states. This study is among the first to combine both objective and subjective assessments of spontaneous blink rate, providing a novel, multi-level perspective on stress reactivity that integrates behavioral and self-reported indicators. Overall, the study emphasizes the mediating role of perceived stress in linking personality and blinking behavior, thereby offering insights into their physiological and psychological interplay.

## 1. Introduction

The Big-Five personality traits of openness to experience, conscientiousness, extraversion, agreeableness, and neuroticism have long been recognized as a comprehensive framework for understanding human personality (Costa & McCrae, 1992; McCrae & Costa, 2003; McCrae & Costa, 1996; McCrae & John, 1992). These traits have been linked to various psychological and physiological processes, including stress responses, emotional regulation, and health behaviors (DeYoung et al., 2010; Strickhouser et al., 2017). The present study investigates the role of personality in eye-blinking and the potential mediating role of perceived stress in these associations.

While self-report questionnaires, such as the BFI-2, are commonly used to assess personality traits, they are subject to potential biases and inaccuracies due to respondents’ lack of self-awareness or unwillingness to answer honestly. Recent research has explored the use of objective physiological measures, such as eye-tracking data, to detect personality traits (Berkovsky et al., 2019). These methods offer the potential for more robust and unbiased personality assessments by leveraging individuals’ unconscious responses to stimuli.

Eye-tracking methods record subtle ocular behavior, such as fixation duration, saccadic movements, and spontaneous blink frequency, while individuals engage with visual stimuli. These parameters provide reliable physiological indicators of attentional control, arousal, and cognitive effort, which are modulated by enduring personality traits (Jongkees & Colzato, 2016; Maffei & Angrilli, 2019; Nakano et al., 2013; Karson, 1983; Berkovsky et al., 2019). For example, individuals high in conscientiousness often display longer fixation durations and lower blink frequencies, reflecting greater attentional stability and self-regulation, whereas those high in neuroticism tend to show more variable and frequent eye movements, consistent with increased emotional reactivity and vigilance (Berkovsky et al., 2019; Wada, 2025; Lahey, 2009). Such measures therefore offer an objective complement to self-report inventories in assessing the behavioral expression of personality.

It is also important to distinguish between physiological blinks, which primarily lubricate and protect the ocular surface, and psychological or cognitive blinks, which are modulated by attention, information processing, and affective states. Physiological blinks typically occur at regular intervals to maintain the tear film, whereas psychological blinks fluctuate dynamically with cognitive load and emotional engagement (Nakano et al., 2013; Maffei & Angrilli, 2019; Wascher et al., 2014). This distinction clarifies that blinking behavior can act as a peripheral indicator of central nervous system activity, bridging physiological maintenance with higher-order psychological functions.

Beyond its ocular function, spontaneous blink rate (SBR) has been increasingly recognized as a psychophysiological marker linked to dopaminergic, affective, and motivational systems. Empirical research demonstrates that blink rate varies systematically with emotional and cognitive states, including anxiety, depression, and heightened arousal (Karson, 1983; Maffei & Angrilli, 2019). Notably, recent findings suggest that reduced blink rate may serve as a nonverbal indicator of emotional suppression and suicidal ideation, reflecting altered dopaminergic functioning associated with severe affective dysregulation (Joiner et al., 2016). These studies underscore that blinking behavior is not merely an ocular reflex but a behaviorally observable index of underlying emotional and neurobiological processes. Therefore, examining blink rate in the context of personality and perceived stress can provide an integrative view of psychological and physiological stress reactivity.

Finally, the current study conceptualizes the relationship between personality and blinking behavior as indirect, operating through the mediating mechanism of perceived psychological stress. Personality traits such as neuroticism and conscientiousness influence individuals’ stress perception and coping patterns, which in turn affect autonomic and attentional systems that regulate blink frequency (Chen et al., 2022; Segerstrom & Smith, 2019). This mediational perspective provides the theoretical rationale for examining perceived stress as a key pathway linking personality and spontaneous blinking.

### 1.1. Big-Five Personality and Eye Blinking

Spontaneous blink rate (SBR) is a natural physiological behavior that reflects underlying neurobiological and psychological states. It has been linked to dopaminergic activity in the brain and is considered a potential biomarker for neuropsychological functioning (Jongkees & Colzato, 2016). SBR involves automatic eyelid closure without external stimuli, although its neurobiological mechanisms are not yet fully understood (Maffei & Angrilli, 2019).

Recent research suggests that the spinal trigeminal complex may play a role in regulating blinking through interactions with the dopaminergic nigrostriatal system (Kaminer et al., 2011). While the primary function of blinking is to restore the tear film and protect the cornea, spontaneous blink frequency often exceeds the amount necessary for eye hydration (Maffei & Angrilli, 2019).

Beyond ocular maintenance, blink rate appears connected to cognitive processes such as attention, vigilance, and mental effort (Wascher et al., 2014). Studies suggest that blink rate can also be an indicator of “flow experience,” with decreased blink rate, particularly at the onset of engaging tasks, among individuals with certain personality traits (Rau et al., 2017). Technological advancements, such as the use of electrooculography (EOG), enable fine-grained detection of blink patterns and the examination of their association with cognitive workload (Berkovsky et al., 2019). For instance, resting blink rates typically ranging from 10 to 25 blinks per minute decrease to below 5 blinks per minute during tasks requiring intense focus, such as reading (Doughty, 2001). The task type also influences blink patterns, causing blinks to occur at natural pauses and to become synchronized among individuals (Nakano et al., 2009).

It has been suggested that suppressing blinking during demanding tasks helps prevent information loss by maintaining visual continuity. Supporting this, recent studies have shown that during activities like watching videos, blink events are associated with increased activity in the default mode network and decreased activity in the dorsal attention network (Nakano et al., 2013). This indicates that spontaneous blinking involves the inhibition of brain regions responsible for attentional shifts, possibly reflecting attentional engagement during natural perception [for a review, see (Maffei & Angrilli, 2019)].

Several studies have examined the relationship between personality traits and eye movements (Wada, 2025); however, less is known about their associations with eye-blinking. For example, quantitative analyses of blink dynamics have been shown to predict deceptive personality traits during forensic interviews (Gullapalli et al., 2021). One might suggest that personality variables relate to eye blinking rate through their association with psychological stress. For example, highly neurotic individuals tend to exhibit more variable physiological responses to stress, which may influence blinking behaviors (Eysenck & Eysenck, 1985; Norris et al., 2007). Conversely, extraversion, characterized by higher baseline activity levels and social engagement, has been linked to more adaptive physiological regulation (Jackson & Schneider, 2014; Ozer & Benet-Martinez, 2006).

Previous studies indicated that neurotic individuals showed heightened physiological reactivity under stress (Chen et al., 2022; Lahey, 2009). These findings suggest that personality traits might influence eye-blinking patterns, especially under or due to psychological load related to perceived psychological stress.

### 1.2. Perceived Psychological Stress as a Mediator

Perceived psychological stress, conceptualized as an individual’s appraisal of stressors as exceeding their coping capacity, plays a crucial role in physiological responses (Cohen et al., 1983; Lazarus & Folkman, 1984). This role is further supported by meta-analytic evidence, demonstrating a significant relationship between perceived stress and various personality traits (Rau et al., 2017). Elevated stress perception triggers neuroendocrine responses (e.g., HPA axis activation), which can influence autonomic functions, including eye-blink rates (Lupien et al., 2009; Sackeim et al., 1982).

The mediating role of perceived stress has been established in the relationship between personality and physiological outcomes. For example, neuroticism is consistently associated with higher perceived stress levels, which in turn heighten physiological stress responses (Bhatti et al., 2024; Mroczek & Almeida, 2004).

We suggest that individuals with high neuroticism may display increased eye-blink rate variability, mediated by their tendency to heightened perception of stress (Chen et al., 2022; Suls & Martin, 2005). Conversely, traits such as conscientiousness and extraversion might be associated with better stress management and reduced perceived stress (Chen et al., 2022; Penley & Tomaka, 2002), which could contribute to more stable blinking patterns.

### 1.3. Integrative Perspectives

The intersection of personality, stress, and physiological responses aligns with biopsychosocial models of health, emphasizing the role of personality in health-related behaviors and responses (Childs et al., 2014; Engel, 1977). Variations in blinking patterns may serve as peripheral indicators reflecting central nervous system activity influenced by personality and stress perception. Empirical evidence supports the hypothesis that perceived stress mediates the relationship between personality traits, particularly neuroticism, and physiological markers such as blinking (Hagger-Johnson et al., 2012; Schneider, 2004).

Building on this literature, it is reasonable to postulate that personality traits influence eye-blinking patterns through their impact on perceived psychological stress. Specifically, the neuroticism trait, associated with emotional instability and negative affect, may elevate perceived stress, which subsequently affects blinking behavior (Ormel et al., 2013). Conversely, traits such as extraversion and conscientiousness might mitigate perceived stress, leading to distinct blinking patterns (Steel et al., 2008).

Recent advancements in psychophysiological research have highlighted the importance of studying subtle physiological markers, such as eye-blinking behavior, as indicators of psychological processes (Eckstein et al., 2017). These markers offer valuable insights into the complex interplay between personality, stress, and autonomic responses. Moreover, understanding these relationships could have implications for stress management interventions and personalized approaches to mental health (Segerstrom & Smith, 2019).

### 1.4. Hypotheses

Based on the existing literature and theoretical framework, we propose the following hypotheses:

**H1.**  *Neuroticism is positively associated with blink rate, mediated by perceived psychological stress.*

**H2.**  *Extraversion and Conscientiousness are negatively associated with blink variability, mediated by perceived psychological stress.*

**H3.**  *Perceived psychological stress mediates the relationship between Big-Five traits (Neuroticism, Extraversion, and Conscientiousness) and blinking behaviors under stress conditions.*

**Figure 1:**
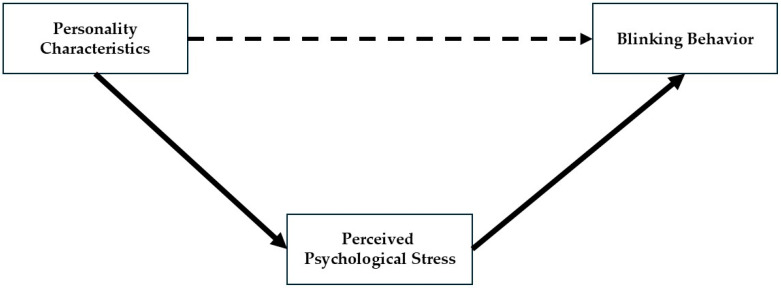
The proposed mediating model depicts the relationship between personality characteristics and blinking responses, mediated by perceived psychological stress. Solid arrows represent the indirect (mediated) paths, whereas the dashed arrow represents the direct (total) effect.

For exploratory purposes, we examined all five traits of the Big-Five personality model. Figure 1 illustrates the proposed model examined in this study, highlighting how personality traits influence blink rate through perceived psychological stress.

Figure 1 illustrates the proposed model examined in this study, highlighting how personality traits influence blink responses through perceived psychological stress.

Understanding how personality traits relate to physiological indicators of stress, such as spontaneous blink rate, may have implications for developing non-invasive markers of psychological functioning. These potential applications are further discussed in Section 4 (the Discussion section).

## 2. Materials and Methods

This cross-sectional prospective study conformed to the ethical principles of the Declaration of Helsinki and was approved by the internal ethics committee of Jerusalem Multidisciplinary College, Israel (JMC, formerly Hadassah Academic College, approval number 0496). The methods were explained verbally, and participants signed a statement of informed consent prior to their participation.

### 2.1. Participants and Procedure

All participants were young adults who spoke and read Hebrew fluently. The final sample included a total of 86 individuals who participated in the study, including 74 females. Their mean age was 22 ± 3 years, with ages ranging from 18 to 31 years.

Participants were recruited via advertisements on the JMC clinic bulletin board, email, and social media. After an oral explanation of the study, participants signed informed-consent forms. Their blinking behavior was recorded while they silently read a standardized Hebrew text: Participants were seated approximately 45 cm from the laptop monitor and instructed to silently read a Hebrew passage displayed in Arial font, size 12, at their habitual reading pace. Participants were informed that their reading would be recorded but, to prevent their awareness of blinking from influencing the results, they were not told that their blink rate would be analyzed. The passage, extracted from a 12th-grade Ministry of Education matriculation exam, consisted of approximately 620 words, 86 sentences, and a total of 5021 characters (with spaces). Silent reading time for the passage is estimated to range between 2 min and 30 s to 3 min at a typical reading speed of 200–250 words per minute. The text covers a detailed description of ecological infrastructure projects along Highway 1 in Israel, focusing on the construction of ecological bridges intended to reconnect fragmented habitats and mitigate environmental harm caused by road expansion. Its language is formal and descriptive, featuring a mixture of technical and environmental terminology, with a lexical density of approximately 61%, reflecting a rich content vocabulary related to ecology, construction, and animal behavior. The tone is neutral and natural to minimize influence on blinking, ensuring that physiological measures accurately reflect engagement with informational content. To avoid confounding effects of any stress induced by the blinking assessment, participants completed an online survey measuring perceived stress and personality traits after a mean of approximately 5 months and the survey was hosted on a secure website.

This interval was intentionally implemented to minimize potential reactivity effects; that is, to prevent participants from consciously linking the physiological assessment with their self-perception of stress or personality, and to accommodate logistical constraints related to academic scheduling and follow-up communication. This delay ensured that the blinking task remained behaviorally natural and unaffected by awareness of subsequent psychological assessment. Participants with a visual acuity worse than 6/9 (20/30) in either eye, active ocular infection, inflammation, past ocular surgery, ocular diseases such as keratoconus, systemic diseases (e.g., hypertension, diabetes mellitus, ischemic heart disease) using medications that might affect the tear film (such as antihistamines and hormones), heavy smokers (more than 20 cigarettes per day), current rigid gas permeable contact lens wearers or those who wore rigid gas permeable lenses for an extended period of two years or more, pregnant or lactating women were also excluded. Inclusion criteria were verified by a certified optometrist (Author RI). Participants with a history of neurological or psychiatric disorders involving abnormal eye movements or motor tics (e.g., Tourette syndrome, blepharospasm, or other movement disorders) were also excluded to ensure that blinking behavior reflected typical physiological functioning rather than clinical or neurogenic conditions.

Although the study was not pre-registered, the data file is publicly accessible on the Open Science Framework (OSF) at: https://osf.io/79ycn/.

### 2.2. Measures

#### 2.2.1. Personality Characteristics

The Big-Five Inventory-2 [BFI-2; (Soto & John, 2017)] is a comprehensive self-report questionnaire designed to assess the Five-Factor Model of personality, consisting of Openness, Conscientiousness, Extraversion, Agreeableness, and Neuroticism. The BFI-2 includes 60 items, with each trait measured by 12 items rated on a 5-point Likert scale from 1 (*strongly disagree*) to 5 (*strongly agree*). The instrument features a hierarchical model with 15 narrower facets, enhancing its bandwidth, fidelity, and predictive power. The BFI-2 has demonstrated robust psychometric properties, including high internal consistency and validity across diverse samples [e.g., (Husain et al., 2025)]. All participants completed the BFI-2 to assess their personality traits, which were used for subsequent statistical analyses. The internal consistency reliability obtained in this study was α = 0.70 for Openness, α = 0.85 for Conscientiousness, α = 0.70 for Extraversion, α = 0.74 for Agreeableness, and α = 0.80 for Neuroticism.

#### 2.2.2. Perceived Psychological Stress

The Perceived Stress Scale-14 [PSS-14; (Cohen et al., 1983)] is a widely used self-report questionnaire designed to measure the degree to which individuals perceive their lives as stressful. It consists of 14 items that inquire about feelings and thoughts related to stress experienced over the past month. Participants rate each item on a 5-point Likert scale from 0 (*never*) to 4 (*very often*), with higher scores indicating higher perceived stress levels. The PSS-14 has demonstrated strong internal consistency, test–retest reliability, and validity across diverse populations (Lee, 2012; Winocur et al., 2011). In the present study, the internal consistency reliability was 0.81.

#### 2.2.3. Subjective Blinking

Participants were asked to indicate their subjective experience related to the rate of blinking. They rated the following one-item scale: “In general, how often do you feel you blink during your daily activities?”, on a scale ranging from 1 (*not at all*) to 7 (*very much*).

#### 2.2.4. Objective Blinking

Objective blinking was recorded while participants silently read a standardized Hebrew text displayed on a Dell Latitude 5440 laptop (Dell Technologies, Round Rock, TX, USA; screen resolution: 1920 × 1080; brightness: 100%) at a mean distance of 45 cm (range: 40–50 cm). Video recordings were captured using the laptop’s built-in camera and stored via the Zoom platform (Zoom Communications, Inc., San Jose, CA, USA; https://www.zoom.com). Each recording lasted approximately five minutes (*M* = 4.928 min, *SD* = 1.143 min).

**Figure 2:**
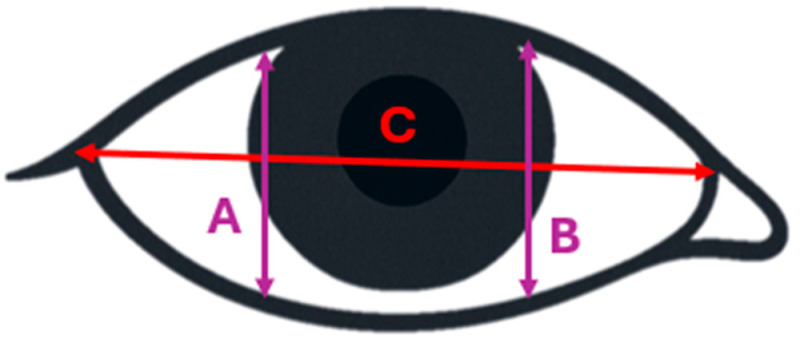
Illustration of the A, B, C parameters in the EAR formula.

*Blink Detection Software*: Custom software was developed using Python 3.14.0 (Python Software Foundation, Beaverton, OR, USA; https://www.python.org) and the dlib 20.0.0 library (https://dlib.net) (Muddada, 2025) with the face recognition package 1.3.0 (face recognition library; https://github.com/ageitgey/face_recognition) [shape-predictor-68-face-landmark]. The software identifies 68 key facial points, with six points used for each eye. An Eye Aspect Ratio (EAR) was calculated using Formula (1):

(1) $$EAR=\frac{A+B}{2C}$$

where A and B represent the vertical eye openings, and C represents the horizontal aperture (Figure 2).

*Blink Detection Algorithm:*

1. Calculate the Mean EAR (mEAR) of both eyes.
2. Establish a threshold for mEAR to detect blinks.
3. Calibrate the threshold individually for each participant.

Threshold Calibration: A personalized calibration was performed for each participant using the first two minutes of their recording. Two clinical professionals (Authors LG and RI) manually counted blinks during this period. The software then employed a Bisection Method (Cormen et al., 2022) to determine the optimal mEAR threshold. *This iterative algorithm narrowed the interval containing the solution by:*

1. Setting initial low and high limits for the threshold.
2. Calculating blinks using the median of these limits as the threshold.
3. Comparing automatic and manual blink counts.
4. Adjusting limits based on the comparison (negative difference: median becomes new high limit; positive: new low limit).
5. Repeating steps 2–4, halving the interval each time until automatic and manual counts converge.

*Data Consideration:*

Once calibrated, the software analyzed the full video recording to determine the mean number of blinks per minute and the standard deviation of the number of blinks per minute. The mean number of blinks per minute served as the physiological outcome measure in the analysis.

To enhance measurement precision, blink duration was calculated in milliseconds (ms) using frame-by-frame temporal resolution from the video recordings (30 frames per second). The Eye Aspect Ratio (EAR) algorithm allowed the identification of the onset and offset of each blink based on the continuous variation in eyelid aperture. The average blink duration across participants ranged between 220 and 380 ms, consistent with established ranges for spontaneous, involuntary blinks (Doughty, 2001). Blinks exceeding 500 ms were automatically excluded to minimize the inclusion of voluntary or prolonged closures that may reflect intentional eye movements or momentary pauses. Although the present analysis focused primarily on spontaneous blinks, we acknowledge that distinguishing between voluntary and involuntary blinking may provide additional insights into how personality traits relate to cognitive versus automatic components of ocular behavior, an issue that could be addressed in future research.

### 2.3. Statistical Analysis

The associations among the study variables were assessed using Pearson correlation coefficients in IBM SPSS Statistics [version 29, (IBM, 2022)]. To test the hypothesized direct and indirect effects of personality traits on eye-blinking rate (blinks per minute), mediated by perceived psychological stress, path analysis was conducted using AMOS [Version 29, (Arbuckle, 2024)]. Statistical significance for all analyses was determined using an alpha level of two-tailed *p* < 0.05.

In addition to assessing statistical significance, the hypothesized mediation path model was estimated using maximum-likelihood path analysis in AMOS. All reported β coefficients represent standardized path coefficients. Because the model was saturated (0 degrees of freedom), global fit indices (e.g., CFI, TLI, RMSEA, SRMR) could not be computed, as the model reproduces the observed covariance matrix perfectly by definition. Gender and age were included as covariates in all analyses; however, controlling for these variables did not alter the pattern or significance of the results and they were thus excluded from further analyses.

## 3. Results

### 3.1. Univariate Analyses: Zero-Order Correlations

A Pearson correlation analysis was conducted on five personality measures, the perceived psychological stress measure, and blink measures (subjective self-report and objective blinks-per-minute). 

The results are presented in the table below (Table 1).

As shown, of the five Big-Five personality traits, two were associated with psychological stress: higher Neuroticism was related to *higher* levels of psychological stress, and higher Conscientiousness was related to *lower* levels of psychological stress. Both of these traits were also marginally associated with subjective reports of blinking (*p* < 0.07); however, only Neuroticism was marginally associated with objective blinking (*p* < 0.07). Openness was the only personality trait significantly associated with reduced objective blinking. In addition, psychological stress was significantly related to objective blinking but not to subjective blinking. Finally, there was no significant correlation between objective blink rate and subjectively reported blinking.

### 3.2. Multivariate Analyses: Direct and Indirect Associations Path Models

To estimate the simultaneous direct and indirect effects of personality characteristics on objective blinking (blinks per minute), a multivariate Path Analysis model with AMOS [Version 29, (Arbuckle, 2024)] was applied using the maximum-likelihood method.

**Figure 3:**
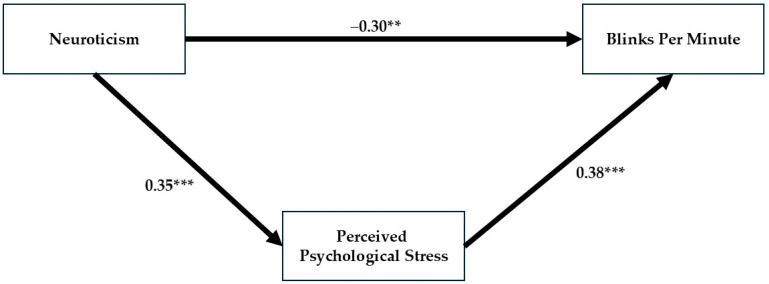
Path model illustrating the direct and indirect effects of Neuroticism on blink rate through perceived psychological stress. Higher Neuroticism predicted higher perceived stress, which was positively related to blink rate, revealing an indirect positive pathway alongside a suppressed direct negative association. Note. ** *p* < 0.01, *** *p* < 0.001.

As can be seen in Table 1, Neuroticism has no significant relationship with objective blinking r = 0.17, ns. In the model where both the direct and indirect relationships were evaluated together (see Figure 3), the direct relationship became stronger, reversed its sign and became statistically significant. Neuroticism is associated with a decrease in objective blink rate: β = −0.30, t = −2.81, *p* < 0.005, while covariating for stress levels. Additionally, a significant indirect relationship was found between Neuroticism and frequent blinking through increased psychological stress: Neuroticism was significantly related to psychological stress (β = 0.35, t = 3.43, *p* < 0.0001), which in turn was significantly associated with blinking (β = 0.38, t = 3.53, *p* < 0.0001).

The findings indicate a *suppression effect*, where the non-significant relationship is strengthened and reverses its sign in the presence of the suppressor variable. Conger (1974) [pp. 36–37] offers the standard definition of a suppressor variable [see also (Hamilton, 1987; Sharpe & Roberts, 1997; Tzelgov & Henik, 1991)]: a variable that, when added to a regression model, improves the predictive power of another predictor (or predictors). In this instance, predictive power is judged by the size of the regression coefficient. Consequently, suppression is indicated when the association between an independent and dependent variable grows stronger after a third variable is included (MacKinnon et al., 2000).

It appears that psychological stress suppresses the relationship between personality and blinking. Neuroticism was found to be associated with blinking after accounting for psychological stress, indicating that stress explains part of the blinking behavior beyond the direct effect of Neuroticism. This model significantly explains 12% of the variance in psychological stress and 15% of the variance in blinking.

**Figure 4:**
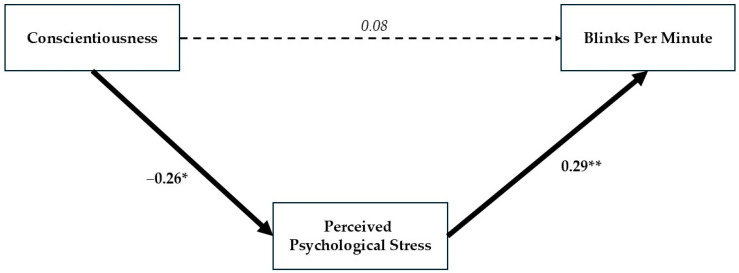
Path model showing the mediating role of perceived psychological stress in the relationship between Conscientiousness and blink rate. Higher Conscientiousness was associated with lower perceived stress, which in turn predicted fewer blinks per minute. Note. * *p* < 0.05; ** *p* < 0.01.

As can be seen in Table 1, Conscientiousness has no significant relationship with objective blinking (r = 0.001). In the model where both the direct and indirect relationships were evaluated together (See Figure 4), Conscientiousness shows no significant direct association with objective blinking: β = 0.08, t = 0.71, *ns*. However, it has a significant indirect relationship with a reduction in objective blink rate through lowering psychological stress: Conscientiousness was significantly associated with lower psychological stress (β = −0.26, t = −2.45, *p* < 0.05), which in turn was significantly related to reduced blinking (β = 0.29, t = 2.71, *p* < 0.01). This model significantly explains approximately 7% of the variance in psychological stress and 8% of the variance in blinking.

## 4. Discussion

This study investigated the relationship between personality traits, perceived psychological stress, and objectively and subjectively perceived eye-blinking behavior in 86 participants. Findings offer valuable insights into the complex interplay between these factors, particularly concerning neuroticism, conscientiousness, and the mediating role of perceived stress. The current findings reinforce the view that blinking behavior reflects an integrated physiological response to psychological stress. Elevated perceived stress was associated with an increased blink rate, consistent with the idea that cognitive and emotional load transiently disrupt attentional stability and enhance autonomic arousal (Nakano et al., 2013; Maffei & Angrilli, 2019). From this perspective, spontaneous blinking can be interpreted as a behavioral marker of momentary fluctuations in stress-related neural activation, particularly within dopaminergic and attention-regulation networks. Importantly, personality traits shape how individuals perceive and respond to stress. Participants high in neuroticism, a trait characterized by emotional instability and heightened stress sensitivity, reported greater perceived stress and exhibited higher blink frequency. In contrast, those high in conscientiousness, associated with organized, goal-directed coping, reported lower stress levels and displayed fewer blinks. These patterns underscore the mediating role of perceived stress as a mechanism through which personality influences physiological stress responses. Thus, blink frequency provides a subtle, non-invasive window into the dynamic interface between personality, stress appraisal, and autonomic regulation.

It is important to note that the present study assessed perceived psychological stress, which reflects individuals’ subjective appraisal of emotional and cognitive strain rather than objective environmental stressors or physiological load. Accordingly, our findings pertain to subjective stress reactivity, that is, how individuals interpret and respond to potentially demanding situations, rather than to actual stress exposure or biological stress indices. This conceptual distinction is particularly relevant given that highly neurotic individuals may appraise everyday situations as more stressful even in the absence of elevated objective demands.

It is also worth noting that participants with neurological or psychiatric conditions associated with abnormal eye movements or motor tics were excluded from the study, ensuring that the observed blinking patterns reflect typical physiological variability rather than clinical or neurogenic disturbances. The relationship between neuroticism and eye-blinking revealed an interesting suppression effect when perceived stress was taken into account. Initially, neuroticism showed no significant direct association with objective blink rate. However, when both direct and indirect relationships were evaluated simultaneously using the path analysis model, a significant negative direct relationship emerged between neuroticism and blink rate, while a positive indirect relationship through perceived stress was also observed.

This suppression effect suggests that the relationship between neuroticism and blinking behavior is more complex than its initial appearance. Specifically, higher neuroticism appears to be associated with decreased blinking when controlling for stress levels. At the same time, neuroticism is linked to increased perceived stress, which in turn is associated with increased blinking. These opposing pathways help explain why the initial zero-order correlation between neuroticism and blinking was non-significant.

It is worth clarifying that the suppression effect observed here is primarily statistical in nature, indicating that the inclusion of perceived stress as a covariate revealed a hidden negative association between neuroticism and blinking. Conceptually, this pattern may reflect two coexisting mechanisms: a direct attentional or inhibitory influence of neuroticism that reduces blinking, and an indirect pathway through heightened stress reactivity that increases blinking. This dual interpretation underscores the complexity of the relationship between personality and physiological responses and helps explain the opposing directions of the observed effects.

Beyond its statistical interpretation, suppression can also be understood in conceptual terms as reflecting the coexistence of opposing psychological processes that act in different directions on the same outcome variable (Tzelgov & Henik, 1991). In the current context, this means that neuroticism may simultaneously promote heightened stress reactivity, leading to more frequent blinking through emotional arousal, while also engaging inhibitory attentional control mechanisms that transiently reduce blinking. Such interplay between facilitative and inhibitory tendencies offers a richer psychological interpretation of the suppression effect, suggesting that the observed statistical pattern may mirror genuine underlying dynamic tensions between emotional reactivity and regulatory control.

While the present analysis focused on mediation pathways linking personality, perceived stress, and blinking, exploratory analyses were also conducted to examine potential moderation effects, in which personality traits might alter the strength or direction of the stress–blinking relationship. However, these Personality × Stress interaction terms did not reach statistical significance and therefore were not retained in the final model. Nevertheless, future research could further explore such moderation effects, as highly neurotic individuals may display an amplified physiological response to stress, whereas highly conscientious individuals may exhibit a dampened association due to greater self-regulatory capacity. Testing these interactions in larger and more diverse samples could extend the theoretical model by capturing differential stress reactivity patterns that complement the mediational processes observed in the current study.

The emergence of this suppression effect highlights the importance of considering mediating variables when examining the relationships between personality traits and physiological responses. It suggests that neuroticism may influence blinking behavior through multiple pathways—a direct pathway that reduces blinking and an indirect pathway mediated by heightened stress that increases blinking. This finding refines our understanding of how personality traits, particularly neuroticism, relate to autonomic responses like blinking.

Results indicate a significant positive association between neuroticism and both perceived stress and objective blink rate. These findings align with previous studies (Bhatti et al., 2024; Chen et al., 2022), suggesting that highly neurotic individuals tend to experience heightened stress levels and exhibit greater physiological reactivity to stressors. These findings are consistent with meta-analytic evidence indicating a strong positive correlation between neuroticism and perceived stress (Rau et al., 2017). The path analysis revealed a significant indirect effect, wherein neuroticism was associated with increased perceived stress, which, in turn, led to an elevated objective blink rate. This supports our hypothesis that perceived stress mediates the relationship between neuroticism and eye-blinking behavior, suggesting that the tendency of neurotic individuals to perceive situations as more stressful contributes to their increased blink rate.

Conversely, we found a negative association between conscientiousness and perceived stress, which is consistent with prior studies (Chen et al., 2022; Penley & Tomaka, 2002) indicating that conscientious individuals tend to employ more effective stress management strategies. This is further supported by meta-analytic findings demonstrating a negative correlation between conscientiousness and perceived stress (Rau et al., 2017). Although the direct effect of conscientiousness on objective blink rate was not significant, the path analysis revealed a significant indirect effect, wherein higher conscientiousness was associated with lower perceived stress, which, in turn, led to a reduced objective blink rate. This suggests that the ability of conscientious individuals to manage stress effectively may contribute to more stable blinking patterns.

Notably, our assessment of blinking behavior employed a dual approach, incorporating both subjective self-report and objective measures. Interestingly, these two blink assessments yielded distinct, uncorrelated results. While the personality measures demonstrated a significant relationship with the objective blink rate, they were unrelated to the subjective self-report of blinking, despite both being self-reported measures. This underscores the importance of utilizing objective measures when examining subtle physiological markers, as subjective reports may be influenced by individual biases or lack of awareness. This methodological approach, which deliberately distinguished between measurement times and methods, strengthens the reliability and validity of our findings, mitigating concerns about concurrent measurement bias. Beyond methodological considerations, the divergence between subjective and objective blink measures may also reflect individual differences in interoceptive awareness, the ability to perceive and interpret internal bodily states. Individuals high in neuroticism, for example, may exhibit heightened self-focus and emotional monitoring that bias their subjective estimates of blinking or stress-related arousal. In contrast, objective blink measures capture automatic physiological processes operating largely outside conscious awareness. From this perspective, the lack of correlation between self-reported and objective indices does not indicate measurement error but rather underscores the multi-level nature of stress reactivity, encompassing both conscious self-perception and implicit autonomic responses (Garfinkel et al., 2015). These distinctions between subjective and objective assessments of blinking are consistent with evidence that fine-grained blink dynamics can index personality-linked tendencies in applied interview settings (Gullapalli et al., 2021). The present study’s findings extend beyond the immediate scope of its hypotheses, offering potential avenues for practical application and further investigation. Given the observed relationship between personality traits, perceived stress, and blinking patterns, our research suggests the possibility of utilizing blink rate as a non-invasive indicator of an individual’s stress level, particularly when considered in the context of their personality profile. This could have implications for the development of objective stress assessment tools, potentially identifying individuals at risk for stress-related health issues. Furthermore, the study highlights the potential for personalized interventions that consider an individual’s personality when designing stress management strategies. For example, individuals with high neuroticism, who are more prone to perceiving stress, might benefit from targeted interventions focused on cognitive reappraisal or emotional regulation techniques. Conversely, individuals with lower conscientiousness could benefit from interventions aimed at improving organizational skills and proactive coping mechanisms. Beyond clinical applications, our findings shed light on the interplay between attention, cognitive load, and physiological responses. Finally, in occupational settings, understanding how personality influences stress reactivity could facilitate the development of tailored employee wellness programs, promoting a healthier and more productive work environment.

While our study provides valuable insights, some limitations should be acknowledged. First, the cross-sectional design limits our ability to infer causality. Future studies should employ longitudinal designs to examine the temporal relationships between personality traits, perceived stress, and blinking. Second, our sample was limited to 86 participants, the majority of whom were female. Future research should include larger and more diverse samples to enhance the generalizability of the findings. Another limitation concerns the exclusive use of self-report measures of perceived stress. Although widely validated, such measures capture subjective appraisals and may not correspond to objective stress exposure or physiological reactivity. Future studies could enhance ecological validity by combining perceived stress ratings with multimodal stress indicators, such as cortisol or autonomic nervous system activity. Third, while we included objective measures of blinking, future studies could incorporate additional physiological measures, such as heart rate variability or cortisol levels, to provide a more comprehensive assessment of stress responses and more precise measurement of blinking through methods like electrooculography (EOG), as demonstrated by (Berkovsky et al., 2019), to distinguish eye-blinking from other biomarkers of stress.

Fourth, future studies could explore other potential mediators and moderators of the relationships between personality, stress, and eye-blinking behavior, such as coping strategies or social support. Additionally, future research could incorporate objective measures of personality, such as those derived from eye-tracking data, to validate the findings based on self-report questionnaires and provide a more comprehensive understanding of the relationship between personality and physiological responses (Berkovsky et al., 2019). Moreover, future research could also investigate the temporal dynamics of flow experiences and their effects on blink rate, as research suggests that the relationship between blink rate and flow may be most pronounced at the beginning of an engaging task, particularly among individuals with dominant or noncompliant personalities (Rau et al., 2017). Finally, future research should explore the potential clinical implications of these findings, such as developing personalized interventions for stress management based on individual personality traits.

## 5. Conclusions

In conclusion, this study provides further evidence for the complex interplay between personality traits, perceived stress, and physiological responses. Neuroticism was positively associated with both perceived stress and objective blink rate, while conscientiousness was negatively associated with perceived stress. These findings highlight the importance of considering personality traits when examining stress responses and suggest that perceived stress may mediate the relationship between personality and blinking behavior. These findings have implications for understanding stress-related behaviors and developing personalized interventions for stress management. By elucidating the complex relationships between personality, perceived stress, and physiological responses, this study contributes to a more nuanced understanding of the interplay between psychological and physiological processes, potentially opening new avenues for research and practical applications in psychology and related fields. Beyond their theoretical significance, these findings also have potential applied implications. Spontaneous blink rate could serve as a non-invasive physiological indicator of stress reactivity, particularly when interpreted within the framework of individual personality differences. Such measures may ultimately inform the design of digital health monitoring systems or occupational stress screening tools that incorporate subtle behavioral markers for early detection and personalized intervention.

## Figures and Tables

**Table 1:** *M*, *SD*, *Skewness*, *Kurtosis* and the zero-order correlation coefficients that personality traits had with psychological stress, subjective and objective blink rate.

	Psychological Stress	Subjective Blinking	Objective Blinking	*M*	*SD*	*Skewness*	*Kurtosis*
Extraversion	−0.033	−0.17	0.05	3.41	0.44	0.20	0.94
Agreeableness	−0.16	0.03	−0.07	3.80	0.49	−0.14	−0.90
Conscientiousness	−0.26 *	−0.20 ^	0.001	3.97	0.57	−0.26	−0.48
Negative Emotionality (Neuroticism)	0.35 **	0.19 ^	−0.17	2.72	0.56	0.71	0.37
Open Mindedness	−0.10	−0.07	−0.21 *	3.49	0.39	−0.15	0.14
Subjective Blinking	0.08	---					
Objective Blinking	0.27 *	0.10	---				
*M*	21.76	4.00	15.81				
*SD*	7.05	1.47	10.07				
*Skewness*	0.17	0.25	1.05				
*Kurtosis*	0.43	0.07	1.41				

^ *p* < 0.07, * *p* < 0.05; ** *p* < 0.001.

## Data Availability

The data presented in this study are openly available in https://osf.io/79ycn/.
